# Association of Wnt Inhibitors, Bone Mineral Density and Lifestyle Parameters in Women with Breast Cancer Treated with Anastrozole Therapy

**DOI:** 10.3390/jcm7090287

**Published:** 2018-09-17

**Authors:** Kristina Bojanić, Ines Bilić Ćurčić, Lucija Kuna, Tomislav Kizivat, Robert Smolic, Nikola Raguž Lučić, Kristina Kralik, Vatroslav Šerić, Gordana Ivanac, Sandra Tucak-Zorić, Aleksandar Včev, Martina Smolić

**Affiliations:** 1 Department of Mineral Metabolism, Faculty of Medicine Osijek, J. J. Strossmayer University of Osijek, Osijek 31000, Croatia; kristina.bojanic@dzo.hr (K.B.); ibcurcic@mefos.hr (I.B.Ć.); tomislavkizivat@gmail.com (T.K.); rsmolic@mefos.hr (R.S.); nikolarlucic@gmail.com (N.R.L.); vatroslav.seric@mefos.hr (V.Š.); atucak@mefos.hr (S.T.-Z.); avcev@mefos.hr (A.V.); 2 Department of Radiology, Health Center Osijek, Osijek31000, Croatia; 3 Department of Pharmacology, Faculty of Medicine Osijek, J. J. Strossmayer University of Osijek, Osijek 31000, Croatia; 4 Department of Medicine, University Hospital Osijek, Osijek 31000, Croatia; 5 Department of Biology, Faculty of Dental Medicine and Health Osijek, J. J. Strossmayer University of Osijek, Osijek 31000, Croatia; lucija.kuna@fdmz.hr; 6 Department of Chemistry, Faculty of Dental Medicine and Health Osijek, J. J. Strossmayer University of Osijek, Osijek 31000, Croatia; 7 Clinical Institute for Nuclear Medicine and Radiation Safety, University Hospital Osijek, Osijek 31000, Croatia; 8 Department of Pharmacology, Faculty of Dental Medicine and Health Osijek, J. J. Strossmayer University of Osijek, Osijek 31000, Croatia; 9 Department of Medical Statistics and Medical Informatics, Faculty of Medicine Osijek, J. J. Strossmayer University of Osijek, Osijek 31000, Croatia; kristina.kralik@mefos.hr; 10 Department of Diagnostic and Interventional Radiology, University Hospital Dubrava, Zagreb 10000, Croatia; gordana.augustan@gmail.com; 11 Department of Pathophysiology, Faculty of Dental Medicine and Health Osijek, J. J. Strossmayer University of Osijek, Osijek 31000, Croatia

**Keywords:** aromatase inhibitors, bone density, breast neoplasms, Wnt signaling pathway

## Abstract

Aim: To determine the levels of Wnt inhibitors in patients treated with aromatase inhibitors (AIs) prior to therapy and to investigate their association with bone mineral density (BMD) and lifestyle parameters. Methods: 137 breast cancer patients were divided into a group treated with 1 mg of anastrozole and a group w/o anastrozole therapy. Serum concentrations of sclerostin and dickkopf1 (DKK1) were measured by ELISA. BMD was measured by dual-energy X-ray absorptiometry (DXA). Lifestyle factors were investigated by a self-reported questionnaire. Results: Sclerostin was significantly higher in the AI-treated group (31.8 pmol/L vs. 24.1 pmol/L; *p* < 0.001), whereas DKK1 was significantly lower in the AI-treated group (24.3 pmol/L vs. 26.02 pmol/L; *p* < 0.001). Total hip and femoral neck BMD were significantly lower in the AI-treated group. Conclusion: AI treatment was associated with increased levels of sclerostin and decreased levels of DKK1.

## 1. Introduction

The treatment of breast cancer (BC) is multidisciplinary and includes surgery, radiation, chemotherapy and adjuvant endocrine therapy in cases of hormone receptor-positive BC. Two-thirds of all BCs are estrogen receptor (ER)-positive and, thus, are candidates for either selective ER modulators (SERMs) or third generation aromatase inhibitors (AIs). AIs (anastrozole, letrozole and exemestane) have demonstrated superior efficacy, and better overall safety in the adjuvant treatment of women with ER-positive BC, compared with the SERM tamoxifen [1,2]. At least eight major clinical trials indicated that anastrozole alone is associated with a longer disease-free survival than therapy with tamoxifen alone [3] which supports the use of AIs as a first-line therapy or as a second-line therapy after treatment with tamoxifen [4] according to the American Society of Clinical Oncology recommendations in their clinical practice guidelines in 2010 and in 2014 [4,5]. However, AIs reduce the conversion of steroids to estrogen, and consequently lower estrogen levels, increase bone turnover and result in the loss of bone mass [6,7]. According to the recent study aromatase inhibitor-associated bone loss (AIBL) occurs at a rate at least 2-fold higher than bone loss seen in healthy, age-matched postmenopausal women, resulting in a significantly higher fracture incidence. It is concluded that adjuvant AI therapy depletes residual estrogen and is associated with rapid bone loss, and the increased fracture risk is distinctly different from that observed in postmenopausal osteoporosis [7]. Similar findings in the ATAC (Arimidex, Tamoxifen, alone or in combination) bone subprotocol confirmed that adjuvant anastrozole therapy for postmenopausal women with early breast cancer led to accelerated bone loss [8]. 

Bone turnover is a physiological process tightly controlled by a complex system of signaling pathways regulating both osteoclast and osteoblast function. While osteoclastic differentiation and activity is regulated by RANKL-OPG signaling pathway [9], Wingless-type (Wnt) signaling is one of the main regulators of osteoblast activity. Wnt ligand binding to its receptor promotes osteoblast differentiation [10]. Activation of this bone anabolic pathway is prevented by Wnt inhibitors such as sclerostin and dickkopf1 (DKK1). 

Sclerostin as a product of the *SOST* gene is a glycoprotein secreted by mature osteocytes [11]. As a potent Wnt inhibitor, it has an anti-anabolic role. Apart from being an antagonist of the Wnt signaling pathway, sclerostin has catabolic activity through a RANKL-dependent pathway. Increasing osteocytic expression of receptor activator of nuclear factor kappa B ligand (RANKL) consequently increases osteoclast production [12]. While sclerostin is almost exclusively expressed in osteocytes [13], DKK1 is secreted by a variety of different cells and tissues including cancer cells. In addition to its direct inhibitory effect of on osteoblasts, DKK1 disrupts the Wnt3a-regulated osteoprotegerin and RANKL expression in osteoblasts and, thus, indirectly enhances osteoclast function in multiple myeloma patients [14]. 

The negative impact of AIs on bone health is well known and proven while the role of Wnt inhibitors in AI-induced bone loss is unknown and still being investigated.

Therefore, this cross-sectional study was designed to determine the circulating levels of Wnt inhibitors in patients with BC treated with AIs and to compare them with a group of patients suffering from BC before AIs therapy. The value of these, as prognostic markers of AI-induced undesirable side effects was investigated as well. Additionally, we aimed to correlate levels of Wnt inhibitors with bone mineral density (BMD) measurements and with lifestyle parameters of participants.

## 2. Methods

### 2.1. Patients

The study protocol was approved by the Ethical Committee of the Health Center Osijek (Ethical Approval Code: 08-1621-1/14), Ethical Committee of the Clinical Hospital Center Osijek (Ethical Approval Code: R2-1099-1/2016) and by the Ethical Committee for Research at the University J.J.Strossmayer, Faculty of Medicine Osijek (Ethical Approval Code: 2158-61-07-15-128). All participants signed an informed consent form before being included in the study.

One hundred thirty-seven postmenopausal patients with ER-positive BC were included in the study from September 2015 to February 2017. For the observation of major differences with the significance level of 0.05 and 80% test strength, a total of 85 subjects are needed to be included in the study according to G*Power ver. 3.1.2 analysis program [15]. All women were examined at the Department of Ultrasound Diagnostics and Mammography at the Health Center Osijek. Participants were divided into a group of postmenopausal patients with BC treated with 1 mg of anastrozole, and a group of postmenopausal patients before anastrozole therapy. All participants were treated according to the standard guidelines.

**Figure 1:**
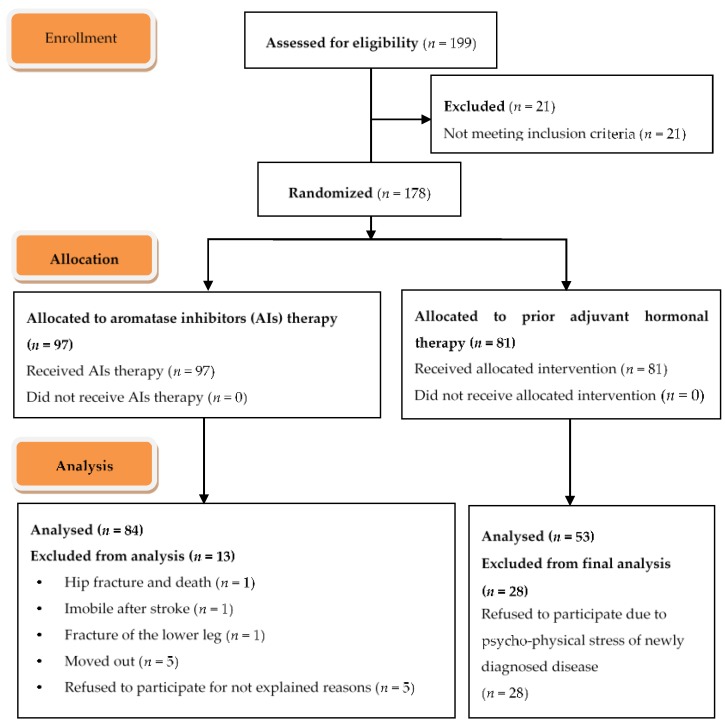
A schematic presentation of flow of study participants from screening to the study endpoint.

Subjects were excluded if they had a history of prior osteoporosis treatment or if they received treatment with medications that interfered with bone metabolism such as chronic corticosteroids (>3 months duration). They were also excluded if they had a history of eating disorder, primary hyperparathyroidism, untreated hyperthyroidism, chronic kidney, gastrointestinal or liver disease. A flowchart of study subjects from assessment for eligibility to final analysis is shown in Figure 1.

A self-reported questionnaire was used to investigate the association of age, anthropometrical parameters and lifestyle factors with other determinants of the study.

After medical interview, serum was obtained between 8 a.m. and 10 a.m. from participants after an overnight fasting and was stored at −80 °C until assayed. In the group of AI-treated patients sampling was done 6 months after AI therapy was initiated. 

### 2.2. Sclerostin ELISA

Serum sclerostin concentrations were measured using a human sclerostin ELISA kit (Biomedica Co., Wien, Austria) according to the manufacturer’s instructions. Intraassay and interassay CVs were 5% and 3–6%, respectively. Briefly, 20 μL of serum were applied as determined by pretesting. Plates were prepared as recommended, serum and all reagents were exposed at room temperature (18 °C–24 °C) before use in the assay. Samples and controls in plates were treated with sclerostin antibody and incubated overnight at room temperature in the dark. The following day, plates were washed and incubated with conjugate for 1 h at room temperature in the dark. After washing, the substrate was added for 30 min. Finally, stop solution was added and values were calculated as instructed. Absorbance was measured at 450 nm with reference of 630 nm, and measurements were reported in pmol/L with a lower limit of detection of 7.5 pmol/L.

### 2.3. Dickkopf1 ELISA

Serum concentrations of DKK1 were measured by DKK1 ELISA kit (Biomedica Co., Vienna, Austria) intraassay CV 3%; interassay CV 3%. Briefly, 20 μL of undiluted serum samples were applied to the relevant wells, and biotinylated anti-DKK1 antibody was applied. Following incubation of 2 h at room temperature in the dark, wells were washed and conjugate was added for an additional one hour. Subsequently, the substrate was added and absorbance was measured after an incubation period of 30 min after the reaction was stopped using the stop solution. Calculation of values was conducted as proposed by the manufacturer.

#### Assessment of Bone Mineral Density

Bone mineral density (BMD; g/cm^2^) was measured by dual-energy X-ray absorptiometry (DXA) imaging (Lunar Prodigy, GE Healthcare, SAD) at the lumbar spine, total hip and femoral neck. Lumbar spine BMD was determined using the anteroposterior projection and was calculated as the average of L1–L4. Both hips and proximal femur scans were used and mean values were calculated. In both groups BMD was measured at the baseline; in the case group at any time during anastrozole treatment, and in the control group prior to anastrozole treatment. BMD values were standardized as T-scores by the same operator according to the operating procedures of the manufacturer. The performance characteristics and standardized quality control procedures of these methods have been described previously [16].

### 2.4. Statistical Analysis

The Mann–Whitney *U* test was used to compare the median between the two groups, analysis of covariance was used in examining the differences in the mean values of the dependent variables that are related to the effect of the controlled independent variables (adjusted for smoking, vitamin D intake, exercise and exercise in the youth), and Fisher’s exact test was used to analyze the differences between proportions compare samples. Pairwise comparisons were used with Bonferroni corrections. The Spearman’s Rho test was used to determine the association between non-normally distributed variables. The level of significance was set at alpha of 0.05. The statistical analysis was performed using MedCalc Statistical Software version 18.2.1 (MedCalc Software bvba, Ostend, Belgium; http://www.medcalc.org; 2018).

The primary endpoint of this study was to determine the circulating levels of Wnt inhibitors in patients with BC treated with IAs, and to compare them with a group of patients with BC before introduction of AIs therapy. The secondary endpoints included correlations of levels of Wnt inhibitors with BMD, lifestyle parameters and eating habits in order to investigate the value of Wnt inhibitors as potential markers of the development and variability of undesirable AIs induced side effects. 

## 3. Results

The study included 137 postmenopausal women diagnosed with hormone receptor (ER) positive BC. 84 postmenopausal patients on therapy with AIs (61.3%) had a mean age of 64 years (interquartile range IQR 56–70 years), and a group of 53 patients (38.7%) w/o AIs therapy had mean age of 59.5 years (IQR 55–67 years). There were no significant differences between the two groups in their age, height, height in youth, weight and BMI as shown in Table 1. There were no significant differences between the two groups regarding lifestyle and eating habits such as alcohol consumption, calcium intake, consumption of dairy products or previous hip fracture. Patients in the AI-treated group smoked significantly more, and had a higher vitamin D intake for up to one year. Women in group w/o AIs were more concerned with physical activity at the time of conducting research, as well as earlier in youth, and the majority of women exercised four and more times a week. There were no significant differences in the age of the first menstrual period, the age of menopause between the groups and a large majority of participants had regular cycles (Table 1).

### 3.1. Primary Endpoint

**Figure 2:**
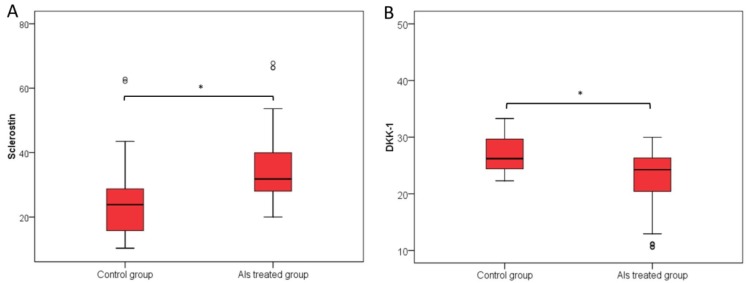
Circulating levels of sclerostin (Panel **A**) and DKK1 (Panel **B**) in AI-treated group and in the group of patients w/o AIs therapy. (Median, interquartile range). Significantly higher sclerostin values were in the AI-treated group of patients, with median 31.8 (IQR 28 to 40.2) compared to the group w/o AIs therapy, with median 24.1 (IQR 15.2 to 28.9) (Mann Whitney *U* test, *p* < 0.001) (Panel **A**). DKK1 was significantly lower in the AI-treated group, with median 24.3 (IQR 20.4 to 26.4) compared to the median of patients w/o AIs therapy 26.02 (IQR 24.1 to 29.9) (Mann Whitney *U* test, *p* < 0.001) (Panel **B**). * Mann Whitney *U* test.

Serum sclerostin levels were significantly higher in the group of postmenopausal patients treated with anastrozol (31.8 pmol/L) compared with the group of patients w/o AIs therapy (24.1 pmol/L; *p* < 0.001) as shown in Figure 1. In contrast, serum levels of DKK1 were significantly lower in the AI-treated group (24.3 pmol/L vs. 26.02 pmol/L; *p* < 0.001) as shown in Figure 2.

### 3.2. Secondary Endpoints

**Figure 3:**
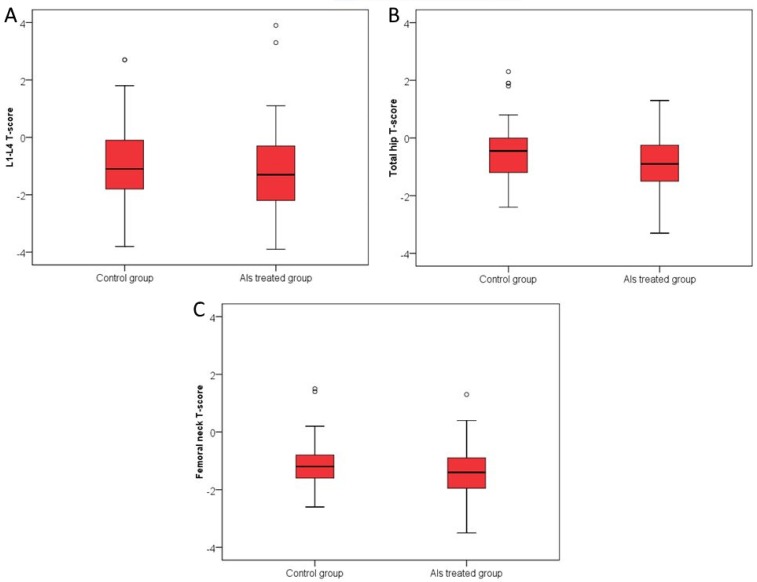
*T-Score* values in AI-treated group and in the group of patients w/o AIs therapy: L1-L4 T- Score (Panel **A**); Total hip *T*-Score, AI-treated group vs. group w/o AIs therapy *p* = 0.01 (Panel **B**); Femoral neck T-Score, AI-treated group vs. group w/o AIs therapy *p* = 0.03 (Panel **C**) (Median, interquartile range). −1.3 (IQR −2.2 to −0.3) was the *T*-Score of lumbar spine in the AI-treated group of patients, −1.1 (IQR −1.8 to −0.1) was the value in the group of patients w/o AIs therapy, without significant difference between the two groups. There is a significant difference in the total hip *T*-Score values between the groups with the median −0.9 (IQR of −1.5 to −0.23) in the AI-treated group, and the median of −0.45 (IQR −1.18 to 0) in the group of patients w/o AIs therapy (Mann Whitney *U* test, *p* = 0.01). Significantly lower femoral neck *T*-Score values in the AI-treated group of patients with median −1.4 (IQR −2 to −0.9) compared to the median of a group w/o AIs therapy −1.2 (IQR −1.6 to −0.8) (Mann Whitney *U* test, *p* = 0.03).

Total hip and femoral neck BMD were significantly lower in the AI-treated group than in the group of patients w/o AIs therapy (−0.9 vs. −0.45 g/cm²; −1.4 vs. −1.2 g/cm²; AI-treated group vs. group w/o AIs therapy; *p* < 0.05) while there were no significant differences in lumbar spine BMD between the two groups as shown in Figure 3.

**Figure 4:**
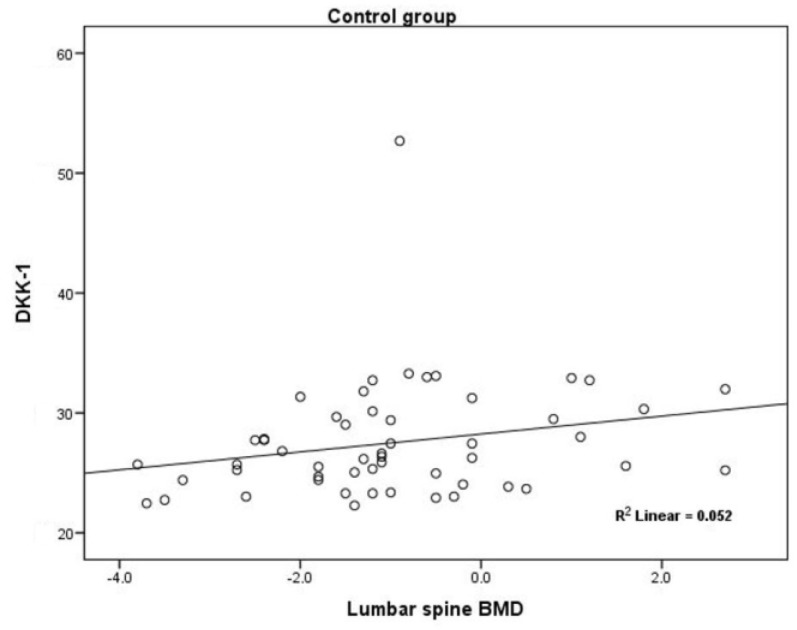
Positive Spearman’s Rho (ρ) correlation between serum DKK1 levels and lumbar spine *T*-Score (Rho = 0.291; *p* = 0.03).

A positive association existed between serum DKK1 levels and lumbar spine BMD (Rho = 0.291; *p* < 0.03), but only in the group of patients w/o AIs therapy (Figure 4). No significant correlations were observed between circulating levels of Wnt inhibitors and BMD measurements in the AI-treated group.

**Figure 5:**
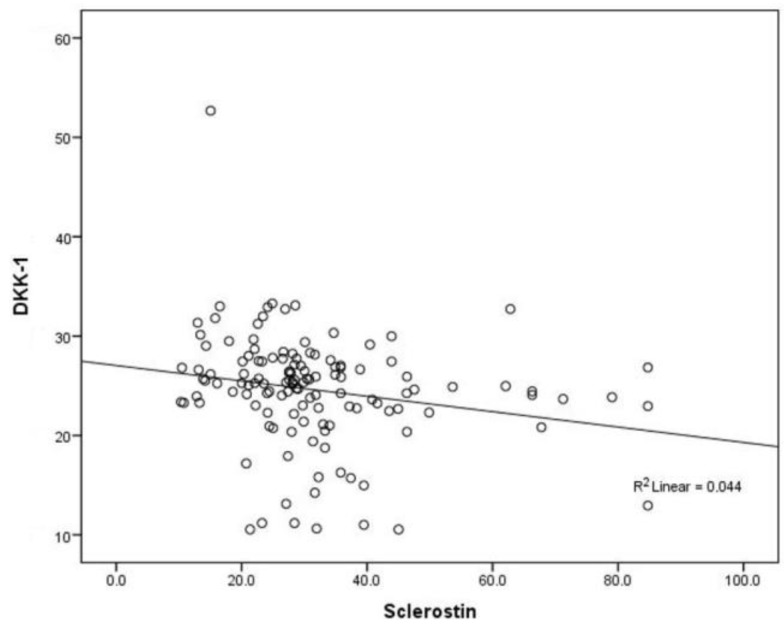
Negative Spearman’s (Rho) correlation between serum sclerostin and DKK1 levels (Rho = −0.287; *p* < 0.001).

Statistically significant negative correlation between sclerostin and DKK1 (Rho = −0.287; *p* < 0.001) was observed when all patients were merged into one group of postmenopausal women with ER positive BC (Figure 5).

When comparing sclerostin and DKK1 levels between AI-treated and AIs non-treated group of patients with regard to lifestyle parameters, significant differences in most variables were observed, suggesting that the relation between AI treatment and sclerostin is not influenced by the lifestyle factors. The exception was presence of regular menstrual cycles which could be identified as potential factors affecting serum levels of sclerostin. The subjects who had regular menstrual cycles had significantly higher values of sclerostin in the AI-treated group compared to the group of patients w/o AIs therapy (Table 2). In addition to regular menstrual cycles two other parameters, no calcium intake and intake of dairy products, affected reduction of DKK1 levels (Table 3).

## 4. Discussion

To our knowledge, this is the first cross sectional study investigating the effects of AIs on circulating levels of sclerostin and DKK1 in postmenopausal women with ER-positive BC before and during adjuvant therapy and exploring their associations with BMD and lifestyle parameters.

In the current study, there were significantly higher sclerostin levels in the group of postmenopausal patients treated with anastrozole compared to the group of patients prior anastrozole therapy. These data are in accordance to other recent studies confirming that sclerostin is a prominent negative regulator of bone formation, which works by impairing osteoblast differentiation and activity [10,17]. Therefore, sclerostin may be a potential specific marker for bone metabolism and assessment of skeletal status. Serum sclerostin levels are regulated by estrogens in postmenopausal women, and a significant negative relationship between serum sclerostin levels and free estrogen index was found in the recent study by Mirza et al. [18]. Our results are in accordance with a previously mentioned study, and could be explained by the fact that AIs ablate any remaining estrogen [18]. The same results have been confirmed by Wonjin at al. who suggested that sclerostin may be a marker for quantitative and sensitive changes in estrogens and bone remodeling [6].

Total hip and femoral neck BMD were significantly lower in the AI-treated group of patients compared to the group w/o AIs therapy, supporting the hypothesis that rapid and sustained increase of sclerostin causes an increase in bone metabolism and subsequently results in BMD loss [19]. However, some studies have shown a positive correlation between serum sclerostin levels and BMD measurements [6,7,20,21]. The same was not observed in the current study.

Significantly lower serum levels of DKK1 in the AI-treated group of patients were found in the current study. These data are in accordance with the results of Kyvernitakis et al. who investigated the effects of aromatase inhibition on circulating levels of Wnt inhibitors in postmenopausal women with ER-positive BC without a control group. In that study, serum DKK1 levels modestly decreased and DKK1 presented modest negative associations with the BMD of the femoral neck and the total hip after 24 months of anastrozole treatment [17]. These results were not confirmed by Gobel et al. who investigated effects of adjuvant AIs treatment on DKK1. They did not find significant differences between DKK1 serum levels at any time points during the study [22]. In our group of patients on AIs therapy, serum levels of DKK1 were measured after the surgical removal of the breast cancer and during AIs adjuvant treatment, while in the control group of patients they were measured prior to the treatment. Therefore, our results could be explained by the fact that breast cancer cells produce high amounts of DKK1 [23,24] and it might be possible that DKK1 levels differ in patients depending on disease status. Besides, the ELISA used in the study of Gobel and as well in the current study does not distinguish between non-tumor and tumor-derived DKK1. In the study by Voorzangler-Rousselot et al., higher DKK1 levels were found in women with BC and bone metastases compared to women with BC in complete remission, women with BC and metastases at non-bone sites and healthy women [23]. Also higher DKK1 serum levels were established in postmenopausal women with osteoporosis without BC [24]. Future studies focusing on comparative evaluation of serum DKK1 and sclerostin levels in women with and without BC might be useful to address this issue.

A positive association between serum DKK1 levels and lumbar spine BMD (Rho = 0.291; *p* < 0.03) was found in the current study as one of secondary endpoints, but only in the group of patients w/o AIs therapy. As previously mentioned, Kyvernitakis et al. found a negative association of DKK1 with BMD in AI-treated group of postmenopausal women with ER-positive BC [17]. The similar results were showed in the studies by Butler et al. and Gatti and et al. [24,25], however this was not confirmed in our study. Therefore, our results could be explained by the fact that women w/o AIs therapy had higher serum levels of DKK1 compared to a AI-treated group of patients, but there were no significant differences in lumbar spine BMD between these two groups. Further investigations of these relationships in larger prospective studies are needed. 

When analyzing entire group of patients, a statistically significant negative correlation between sclerostin and DKK1 (Rho = −0.287; *p* < 0.001) was found (Figure 4). In the literature, there are some studies with contrary results of the relationship between Wnt inhibitors. Different DKK1 and sclerostin results have been previously described in the context of anti-resorptive treatment in postmenopausal women [25]. However, recent studies have not found any association between serum levels of sclerostin and DKK1 [26]. On the contrary, in the study by Sankaralingam et al. who investigated changes in DKK1 and sclerostin following a loading dose of vitamin D2 (300,000 IU), a significant positive correlation between Wnt inhibitors at baseline and after 3 months of high dose vitamin D was found [27]. Authors speculate that the explanation for the observed divergence could be the difference in assays used as they may have measured different regions or fragments of the sclerostin and DKK1 molecule [27]. In the current study, the negative association could reflect different biologic events possibly due to the fact that sclerostin activity is a part of a physiological process of bone remodeling, and is not secreted by cancer cells as opposed to DKK1 which is produced by tumor cells [28,29,30,31]. Therefore, it could be negatively affected by anastrozole therapy resulting in negative association between those two markers.

As possible regulators of circulating DKK1 levels consumption of dairy productsand the presence of regular menstrual cycles were highlighted, that showed significantly lower serum DKK1 levels in the AI-treated group compared to the group w/o AIs therapy. Possible sclerostin regulator isthe presence of regular menstrual cycles that were associated with significantly higher serum sclerostin levels in the AI-treated group. Some studies have shown that serum sclerostin levels were increased by mechanical unloading, immobilization, male sex, age, estrogen deficiency [32,33,34]. However, in the study by Dovjak et al., the differences in serum sclerostin levels between young and geriatric females were not observed. These data are in accordance with our results since no associations between age and serum levels of Wnt inhibitors were observed (data not shown). On contrary, according to the study by Liakou et al. circulating sclerostin and DKK1 levels do not change across the menstrual cycle and do not demonstrate any relationship with estradiol in premenopausal women [35]. To address this issue additional experimental and clinical studies in a larger population are needed.

A limitation of our study could be the cross-sectional design, and the lack of longitudinal data as well as relatively small sample size.

The main distinctive characteristic of our study is the presence of a concurrent group which consisted of subjects enrolled simultaneously with the treatment group from the same source population of BC patients before initiation of AI treatment and compared with patients receiving the AI treatment.

A further advantage is that we had specific inclusion and exclusion criteria for the selection of patients treated with AI and this rigorousness was evident with regards to selection of patients before initiation of AI therapy as well.

## 5. Conclusions

In conclusion, the current data showed that serum sclerostin levels were significantly increased in the AI-treated group of postmenopausal patients with ER-positive BC, while serum levels of DKK1 significantly decreased as a result of AI treatment. Therefore, we conclude that serum levels of Wnt inhibitors may offer new potential markers for assessing skeletal status in AI treated BC patients and could be useful as additional markers to maintain bone health, and to ensure an optimal quality of life of AI treated BC patients.

## Figures and Tables

**Table 1:** Baseline patient characteristics.

	**Median (Interquartile Range)**		*p* *
	**Group w/o AIs Therapy**	**AIs Treated Group**	
Age (year)	62 (55–67)	64 (56–70)	0.06
Hight (cm)	163 (158–168)	162 (158–165)	0.33
Height in youth (cm)	165 (160–169)	164 (160–167)	0.69
Weight (kg)	72 (65.8–82.3)	71 (63–80)	0.48
BMI (kg/m^2^)	27 (24.5–30.9)	27.1 (24–31.2)	0.84
The age of the menopause	50 (45–52)	49 (44–51)	0.17
The age of the first menstrual period	13 (12–14)	13 (12–14)	0.74
	**Number (%) Patients**		***p*^†^**
	**Group w/o AIs Therapy**	**AIs Treated Group**	
Previous hip fracture	0	2 (2.4)	0.52
Previous fracture without trauma	11 (20.8)	11 (13.4)	0.34
Parent with hip fracture	7 (12.7)	6 (7.3)	0.37
Smoking	7 (12.7)	23 (28)	0.04
Alcohol consumption	0	1 (1.2)	>0.99
Vitamin D intake	8 (14.5)	31 (37.8)	0.004
Calcium intake	12 (21.8)	25 (30.5)	0.43
Regular cycles	49 (92.5)	73 (89)	0.57
Physical activity (work in the garden, at home)	18 (32.7)	9 (11)	0.002
Exercise	45 (81.8)	55 (67.1)	0.03
The frequency of exercise			
Once a week	7 (16)	14 (26)	0.16
Two times a week	8 (18)	15 (28)	
Three times a week	11 (24)	6 (11)	
Four times a week	19 (42)	18 (34)	
Total	45 (100)	53 (100)	
Exercise earlier in the youth	45 (85)	47 (57)	0.001

* Mann Whitney *U* test; ^†^ Fisher’s exact test; Anastrozole inhibitors (AIs).

**Table 2:** Differences in serum levels of sclerostin according to the examined lifestyle parameters between the AI-treated and AIs non-treated group of patients (adjusted for smoking, vitamin D intake, exercise and exercise in the youth).

Sclerostin (pmol/L)	Mean (Standard Deviation)		*p* *	^†^ Pairwise Comparisons *p*-Value (95% CI)
	Group w/o AIs Therapy	AIs Treated Group		
Calcium intake				
No	26.8 (15.7)	34.66 (13.4)	**0.03**	0.10 (−12.2 to 1.11)
Yes	23.6 (8.9)	38.8 (16.1)	**0.02**	**0.03** (1.21 to 22.9)
Consumption of dairy products				
No	24.7 (9.7)	36.7 (15.4)	**0.03**	**0.03** (1.36 to 21.9)
Yes	26.8 (16.3)	35.4 (13.7)	**0.03**	0.06 (−0.23 to 13.6)
The age of menopause prior 45 years of life				
No	26.2 (14.6)	34.9 (13.7)	0.09	**0.03** (0.68 to 14.30)
Yes	26.0 (14.9)	38.1 (15.3)	0.21	0.10 (−2.17 to 21.97)
Presence of regular menstrual cycles				
No	38.03 (18.9)	31.3 (5.9)	0.89	0.36 (-32.5 to 13.4)
Yes	25.2 (13.8)	36.5 (14.9)	**0.002**	**0.002** (3.57 to 15.69)

* ANCOVA (adjusted for smoking, vitamin D intake, exercise and exercise in the youth); ^†^ Bonferroni corrected.

**Table 3:** Differences in serum levels of DKK1 according to the examined lifestyle parameters between the AI-treated and AIs non-treated group of patients (adjusted for smoking, vitamin D intake, exercise and exercise in the youth).

DKK1 (pmol/L)	Mean (Standard Deviation)		*p* *	^†^ Pairwise Comparisons *p*-Value (95% CI)
	Group w/o AIs Therapy	AIs Treated Group		
Calcium intake				
No	27.5 (5.1)	22.4 (5.4)	**<0.001**	<0.001 (2.47 to 7.36)
Yes	27.4 (3.6)	22.9 (4.9)	0.13	0.01 (1.13 to 8.58)
Consumption of dairy products				
No	28.0 (7.2)	23.5 (5.3)	0.19	0.11 (−0.91 to 8.65)
Yes	27.2 (3.3)	22.0 (5.1)	**<0.001**	<0.001 (3.13 to 7.25)
The age of menopause prior 45 years of life				
No	27.4 (5.2)	22.7 (4.8)	<0.001	<0.001 (2.55 to 7.29)
Yes	27.6 (3.0)	22.1 (6.1)	<0.001	0.02 (0.64 to 7.35)
Presence of regular menstrual cycles				
No	26.3 (3.8)	24.9 (5.5)	0.34	0.71 (−9.29 to 6.69)
Yes	27.5 (4.9)	22.2 (5.1)	**<0.001**	<0.001 (3.30 to 7.52)

* ANCOVA (adjusted for smoking, vitamin D intake, exercise and exercise in the youth); ^†^ Bonferroni corrected.
